# Satisfied patients after shoulder arthrodesis for brachial plexus lesions even after 20 years of follow-up

**DOI:** 10.1007/s00590-018-2152-8

**Published:** 2018-02-16

**Authors:** M. A. J. van der Lingen, S. G. C. J. de Joode, M. G. M. Schotanus, B. Grimm, F. A. van Nie, L. A. W. M. Speth, S. K. Samijo

**Affiliations:** 1 Department of Orthopaedic Surgery and Traumatology, Maxima Medical Center, Veldhoven, The Netherlands; 2 Department of Orthopaedic Surgery and Traumatology, Zuyderland Medical Center, Heerlen, The Netherlands; 3 Department of Neurosurgery, Zuyderland Medical Center, Heerlen, The Netherlands; 4 Department of Rehabilitation, Zuyderland Medical Center, Heerlen, The Netherlands

**Keywords:** Brachial plexus injury, Shoulder arthrodesis, Satisfaction, Shoulder function

## Abstract

**Purpose:**

Patients with an upper brachial plexus lesion can suffer from dysfunction, joint deformities and instability of the shoulder. The goal of this study was to determine pain, shoulder function, patient satisfaction and muscle strength in shoulder arthrodesis in patients with an upper brachial plexus lesion more than 15 years after surgery.

**Methods:**

We retrospectively studied 12 patients with a brachial plexus lesion of mean age 46 years (27–61). At a mean of 19.8 years (15.4–30.3) after shoulder arthrodesis, patient-reported outcome measures (PROMs), range of motion (e.g., active and passive), patient satisfaction, strength of the affected and non-affected side (e.g., maximum isometric strength in Newton in forward and retroflexion, ab- and adduction, internal and external rotation) and position of fusion were obtained. PROMS consisted of the Visual Analogue Scale (VAS; 0–100, 0 being painless) for pain and the Disabilities of the Arm, Shoulder and Hand Score (DASH; 0–100, 0 being the best score) for function.

**Results:**

At latest follow-up, the median VAS pain score was 49 (0–96) and 0 for, respectively, the affected and unaffected side. The DASH was 15 (8–46), meaning a reasonable to good function of the upper extremity. Active and passive retroflexion was significantly different (*p* = 0.028). All subjects stated that in the same situation they would undergo a shoulder arthrodesis again. The unaffected side was significantly stronger in every direction. Arthrodesis showed position of fusion of 31° (12–70) abduction, 20° (10–50) forward flexion and 22° (− 14 to 58) internal rotation. The unaffected side was significantly (*p* ≤ 0.05) stronger in every movement direction.

**Conclusion:**

At a mean of 20 years after shoulder arthrodesis, patients with an upper brachial plexus lesion are still satisfied with a good to moderate functional improvement.

**Level of evidence III:**

A retrospective cohort study.

## Introduction

The severity of brachial plexus injury varies from transient neuropraxia to avulsion-type injuries. Upper brachial plexus lesions affect shoulder function, and lower brachial plexus lesion affect wrist and hand function. Muscle weakness and joint contractures are common in patients with an obstetric brachial plexus lesion. This may result in joint deformities or persisting instability [1–3].

**Fig. 1:**
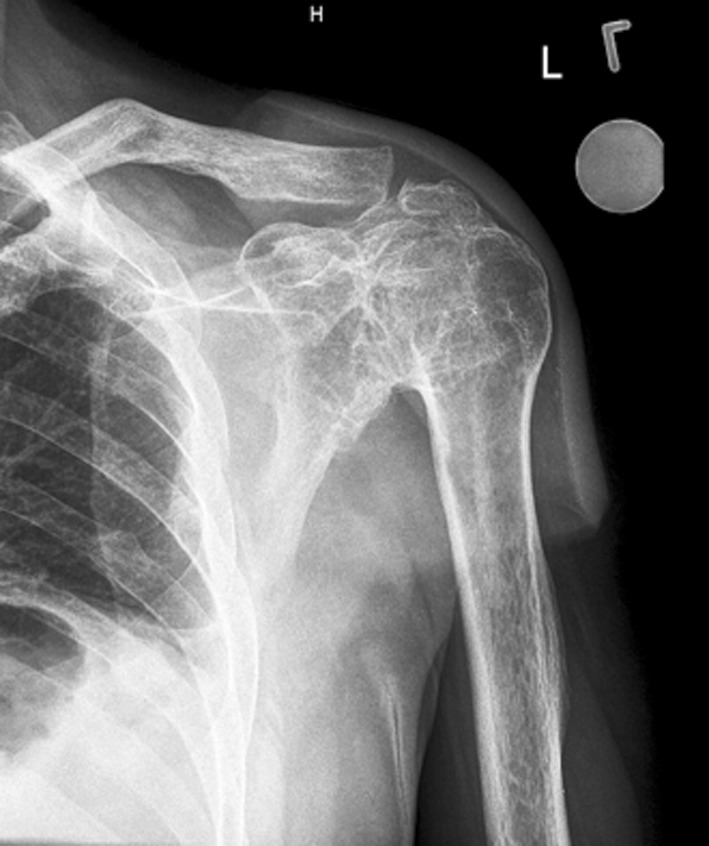
X-ray of the left shoulder of patient 1, 18-years after shoulder arthrodesis

The surgical treatment of traumatic and obstetrical brachial plexus lesions remains challenging [4, 5]. When direct nerve repair and secondary salvage procedures [3, 6] fail to regain satisfactory shoulder function, arthrodesis of the shoulder is viable [7–9]. The main goal is to stabilize the shoulder to optimize elbow and hand function. A good function of the scapular-thoracic joint is a prerequisite to allow some active movement (Fig. 1).

Previous studies regarding shoulder arthrodesis have shown that shoulder arthrodesis substantially results in a decrease in pain during normal daily activities [8, 9] and improves function and muscle strength [10]. The most critical complication of this procedure is malpositioning of the extremity. [11]. The limited number of papers available suggest that hand function is a major determinant of outcome [12]. Chammas et al. found the strength of the pectoralis major a significant prognostic factor as well as recovered elbow flexion [10].

**Fig. 2:**
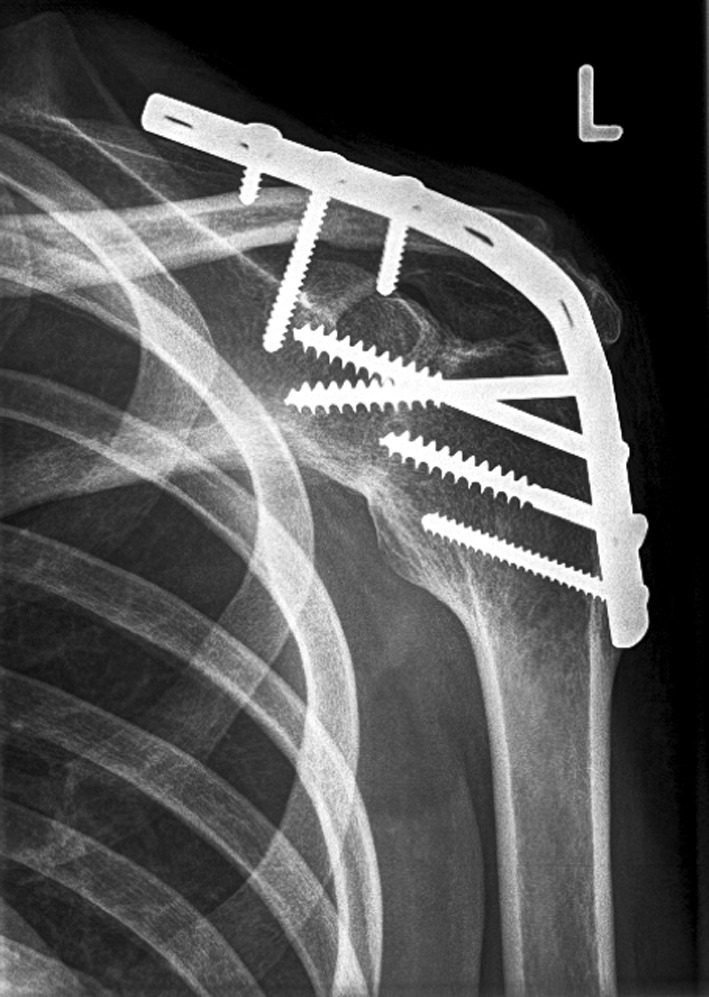
Anterior-posterior X-ray of the left shoulder of patient 12, 15.5-years after shoulder arthrodesis

Since sequelae of brachial plexus injuries evolve at early stage after denervation, it is of importance to document the long-term outcome of shoulder. However, to our best knowledge, long-term follow-up is scarcely reported as the aforementioned studies had a mean follow-up of maximum 15 years [1, 6, 12, 13]. The main goal of this study was to determine pain, shoulder function, patient satisfaction and muscle strength related to the position of fusion in shoulder arthrodesis for brachial plexus lesions more than 15 years after surgery (Fig. 2).

## Materials and methods

A retrospective analysis was made of 12 adults who underwent shoulder arthrodesis for brachial plexus lesions between 1975 and 1990. The cohort consisted of 11 men and 1 woman, with a median age of 46 years (range 27–61) at follow-up. Average follow-up was 19.8 years (15.4–30.3). There was no loss to follow-up. Ten cases were traumatic injuries, and two consisted of obstetrical brachial plexus lesions. Inclusion criteria for shoulder arthrodesis were a normal elbow function and functional levator scapulae, rhomboidei, trapezius and serratus anterior muscles. Patient demographics are summarized in Table 1.

**Table 1:** Patient demographics

Nr.	Gender	Age	Follow-up (year)	Trauma	VAS	DASH	Satisfaction^a^
1.	Male	54	17.4	Yes	0	10	1
2.	Male	36	18.3	Yes	70	16	3
3.	Male	42	17.7	Yes	0	14	1
4.	Male	61	20.8	No	0	8	3
5.	Male	54	30.3	Yes	37	46	1
6.	Male	27	19.4	No	0	21	2
7.	Male	49	16.5	Yes	61	16	1
8.	Male	54	24	Yes	93	31	3
9.	Male	45	20.9	Yes	96	33	3
10.	Female	50	18.5	Yes	12	8	2
11.	Male	41	18.7	Yes	64	11	1
12.	Male	34	15.4	Yes	67	14	1

^a^Satisfaction (1 = highly satisfied, 2 = moderate improvement, 3 = slight improvement, 4 = no improvement)

### Operative technique

**Fig. 3:**
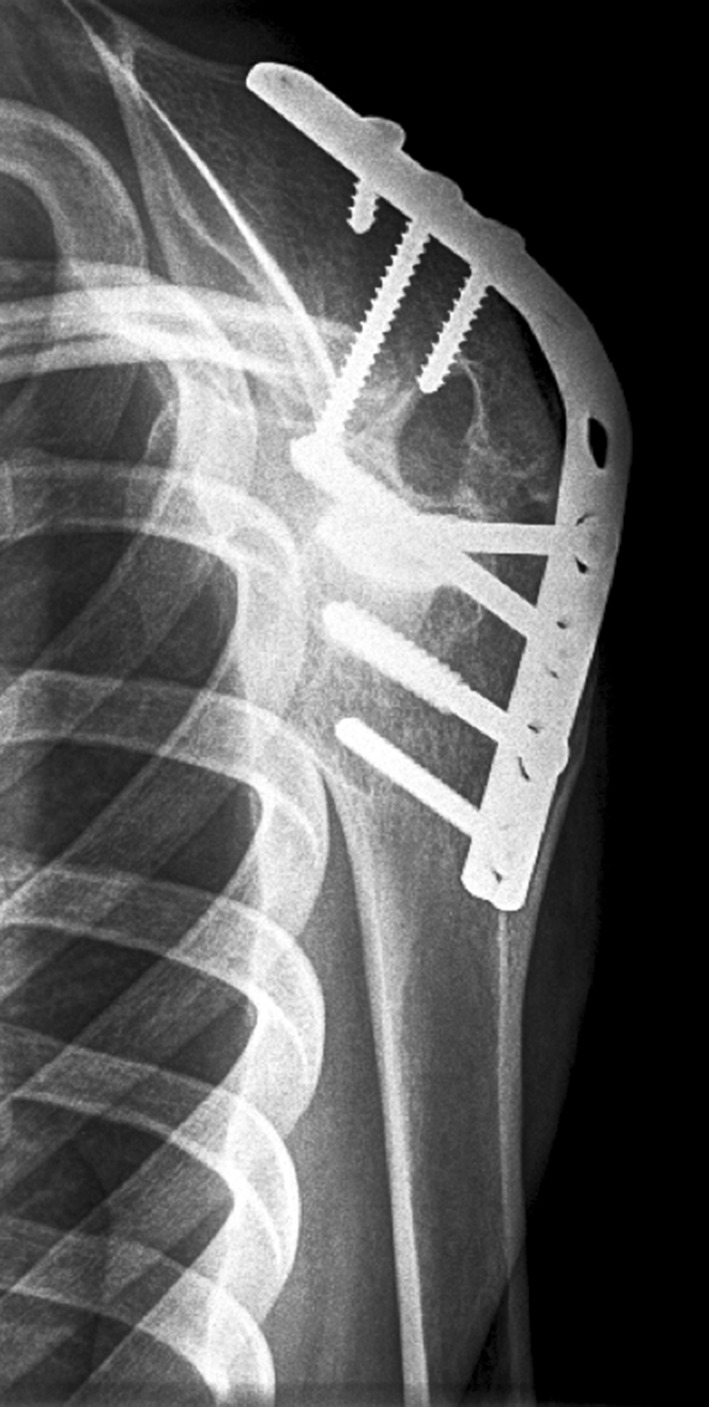
Lateral scapula view of the left shoulder of patient 12, 15.5-years after shoulder arthrodesis

Patients were operated in a lateral decubitus position by one experienced orthopedic surgeon in our brachial plexus specialty service. Fusion angles were aimed according to Rowe: 15°–20° for abduction, 25°–30° for forward flexion and 40°–50° for internal rotation [14]. All arthrodeses were performed using an acromiohumeral AO (*arbeitsgemeinschaft fur osteosynthesefragen*) dynamic compression or reconstruction plate osteosynthesis with lag screw fixation of the humeral head to the glenoid after a complete denudation of the cartilage (Fig. 3).

**Fig. 4:**
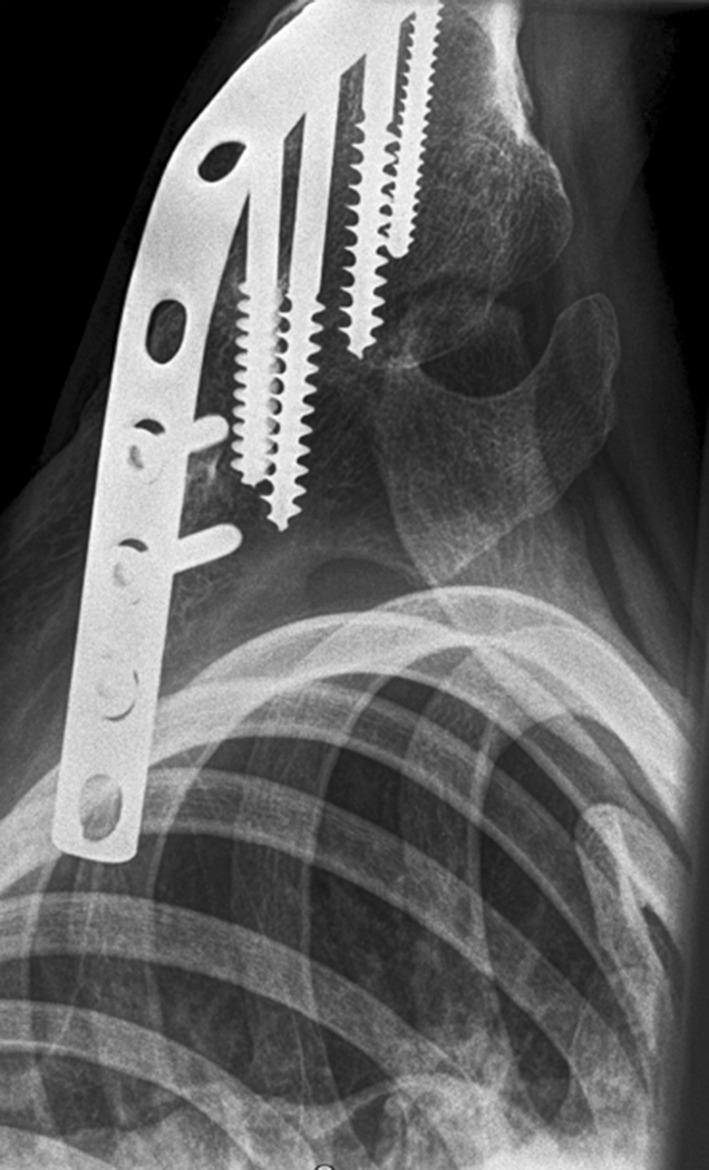
X-ray in axial view of the left shoulder of patient 12, 15.5-years after shoulder arthrodesis

The primary outcome measure was patient-reported pain, measured using the Visual Analogue Scale (VAS); a 100-point scale with 0 indicating no pain and 100 indicating the worst pain imaginary. Secondary outcome measures were: shoulder function, patient satisfaction, muscle strength and position of fusion in degrees (Fig. 4).

Shoulder function was measured using the Disabilities of the Arm, Shoulder and Hand Score (DASH) and the range of motion (ROM) in degrees. The DASH score is a self-report 30-item questionnaire that looks at the ability of a patient to perform certain upper extremity activities. Higher DASH scores indicate a greater level of disability and severity, whereas lower scores indicate a lower level of disability. The score ranges form 0 meaning no disability to 100 meaning most severe disability. Range of motion was measured using a goniometer [15].

Patient satisfaction was recorded using a four-point scale (high, moderate, slight or no improvement) and the question whether subjects would choose a shoulder arthrodesis again.

Muscle strength was measured on the affected and non-affected side as the maximum isometric strength in Newton in forward flexion, retroflexion, abduction, adduction, internal- and external rotation using digital scales (Kern CH50K50, Germany) to which a comfortable handle was attached and which could be adjusted in height for optimum position. The measurement was taken after 5 s because our own pilot study showed that earlier readings fluctuate too much to take an isometric value. Each patient had to perform three times, and the highest value was recorded to exclude submaximal attempts and missed pulling’s. Subjects were instructed to keep an upright position to minimize gravity effects. Patients stood with bare feet on a rough surface to increase friction with the floor and thus optimize force transfer. Alternating individual measurements between the affected and healthy side minimized fatigue.

Position of fusion was determined both clinically and radiographically, on plain anterior–posterior and lateral radiographs as well as by goniometer measurement. Degrees of forward flexion, retroflexion, abduction, adduction, internal and external rotation were measured.

Measurements were taken with the scapula in the anatomical position, symmetrical to the contralateral scapula. For flexion, ab- and adduction the angle between the humerus and the caudal-cranial axis was used. Forward flexion and abduction were described as positive values, and retroflexion and adduction were described as negative values. For rotation, the angle between the humerus and the sagittal axis was used with positive values for internal rotation and negative values for external rotation. Neutral was defined as the non-elevated position of the humerus parallel to the thoracic spine with the forearm in the sagittal plane at 90° elbow flexion. To measure the active range of motion, patients were instructed to perform the movement with maximum effort.

### Statistical analysis

All statistical analysis was done with use of Statistical Package for the Social Sciences version 20.0 for windows (SPSS., Inc., Chicago, IL). Descriptive statistics were used to summarize data. Results are presented as either with median (range) or proportions (%). Significance levels were set at *p* ≤ 0.05.

## Results

The median VAS pain score was 49 (0–96) and 0 (0–44) for, respectively, the affected and unaffected side. The median DASH score was 15 (8–46). The maximum active and passive ranges of motion of the affected arm are summarized in Table 2. Patients have a good function in forward flexion, abduction and internal rotation due to a normal functioning scapular-thoracic joint. Five patients could reach their front pocket, one patient the rear pocket, five the head, seven the mouth with their affected limb, and ten patients could lift an object using both arms.

**Table 2:** Maximum active and passive ranges of motion in degrees of the affected upper arm (deg), relative to the anatomical starting position

	Active		Passive		
	Median	Range	Median	Range	*Δ*
Forward flexion	60	12–72	68	44–94	8
Retroflexion	0	− 24 to 20	10	− 15–30	10
Abduction	48	14–78	64	34–94	16
External rotation	0	− 58 to 30	0	− 58–40	0
Internal rotation	32	6–76	44	18–68	12

Positive values indicate abduction, internal rotation and forward flexion

Negative values indicate adduction, external rotation and retroflexion, relative to the anatomical starting position

Fifty percent of the patients were highly satisfied as two others stated a moderate improvement, while the remaining four patients only experienced a slight improvement. All subjects stated that in the same situation they would undergo a shoulder arthrodesis again.

The isometric strength values are presented in Table 3. As expected, the unaffected side was stronger in every movement direction. The difference (Δ) was largest in abduction.

**Table 3:** Maximum isometric muscle strength in Newton, *Δ* = deviation (deg) between the affected and unaffected side

	Affected side		Unaffected side		
	Median	Range	Median	Range	*Δ*
Forward flexion	42	12–89	73	40–116	31
Retroflexion	18	0–63	75	13–121	57
Abduction	32	0–83	96	33–153	64
Adduction	41	0–121	77	16–164	36
External rotation	30	0–94	69	25–94	39
Internal rotation	36	0–108	73	14–158	37

The positions of the fused glenohumeral joint of the humerus with respect to the scapula are summarized in Table 4.

**Table 4:** Position of fusion in degrees (deg)

	Median	Range
Abduction	31	12–70
Forward flexion	20	10–50
Rotation	22	− 14 to 58

Positive values indicate abduction, internal rotation and forward flexion

Negative values indicate adduction, external rotation and retroflexion, relative to the anatomical starting position

## Discussion

The most important finding of this study is that patients are satisfied with shoulder arthrodesis for brachial plexus lesions even after 20 years of follow-up. Studies reported in the literature could not present a follow-up for longer than 15 years [10].

Pain score after shoulder arthrodesis in this study was similar to values reported earlier [10]. One-third of the subjects reported no pain at all, which is in agreement to the study of Rtaimate et al. where nearly half of the patients were free of pain [7]. In some patients, pain was related to neuropathic pain. The two patients with high VAS scores in this study had a moderate active shoulder function with a good passive function. It seems that external rotation was worse for these patients.

We found out that an important factor for patient satisfaction after operation is a reasonable to good shoulder function. It seems that shoulder function is even more important than pain concerning satisfaction. Patients with better DASH scores are more satisfied. For a good DASH score, a normal functioning scapular-thoracic joint of the affected shoulder and a good function of the unaffected shoulder are necessary. Patients with better DASH scores show higher degrees of active and passive abduction and forward flexion of the affected shoulder. The DASH score measures function based on the ability to perform tasks of daily life. This often requires the use of both arms or can be performed with one arm by a trained disabled person. We advise that therapy following shoulder arthrodesis for plexus lesion should also pay attention to exercises of the unaffected shoulder to maximize the functional outcome.

In this study group, the average position of glenohumeral fusion was 31°, 20° and 22° of abduction, forward flexion and internal rotation, respectively. Position of abduction and forward flexion are in accordance with the literature on shoulder arthrodesis, and the position of internal rotation is lower than that reported [8, 11, 16, 17].

The average fusion angle for internal rotation in our group was lower than the current recommendation of 40°–45° [8, 11, 16, 17]. The literature showed that internal rotation angles exceeding 40° led to dramatically increasing DASH scores [18]. At fusion angles larger than 40°, the shoulder girdle needs more active external rotation for the same movement. Based on these findings, it seems functionally better to fuse internal rotation at slightly lower angles than the current recommendation of 40°. Although our study shows no correlation between lower fusion angles of internal rotation and lower DASH scores, it tends that patients with lower angles of fusion in internal rotation have better shoulder function.

Next to that it tends that increasing angles of forward flexion fusion led to higher levels of pain. It is described that large forward flexion and abduction angles force the scapula to rotate and wing when the shoulder is at rest [17]. This may lead to fatigue of the scapular-thoracic muscles, which could result in pain and irritation.

For the best position of fusion, we advise to make a cast template prior to the operation with the right angles when the patient is sitting up straight. This cast template can be a guidance for the best position during the operation when the patient is lying down in a lateral decubitus position. The chance of errors in the chosen angles is minimized with this technique.

There is a major limitation to the current study. Due to the topic, this study is underpowered, and only 12 patients were included. Larger patient series will be needed to confirm our preliminary results.

## Conclusion

This study demonstrates that shoulder arthrodesis is a suitable operative technique to stabilize the shoulder to optimize elbow and hand function in patients with an upper brachial plexus lesion. At a mean of 20 years after shoulder arthrodesis, patients were still satisfied with a good to moderate functional improvement. Position of fusion is an important factor for shoulder function after shoulder arthrodesis. However, a good function of scapular-thoracic joint in the affected shoulder is even more important.

## References

[1] Hoeksma AF, Ter Steeg AM, Dijkstra P, Nelissen RG, Beelen A, de Jong BA (2003). Shoulder contracture and osseous deformity in obstetrical brachial plexus injuries. J Bone Joint Surg [Am].

[2] Strombeck C, Krumlinde-Sundholm L, Remahl S, Sejersen T (2007). Long-term follow-up of children with obstetric brachial plexus palsy I: functional aspects. Dev Med Child Neurol.

[3] Nath RK, Lyons AB, Melcher SE, Paizi M (2007). Surgical correction of the medial rotation contracture in obstetric brachial plexus palsy. J Bone Joint Surg Br.

[4] Birch R (1993). Surgery for brachial plexus injuries. J Bone Joint Surg Br.

[5] Giuffre JL, Kakar S, Bishop AT, Spinner RJ, Shin AY (2010). Current concepts of the treatment of adult brachial plexus injuries. J Hand Surg Am.

[6] Ruhmann O, Wirth CJ, Gosse F (1999). Secondary operations for improving shoulder function after brachial plexus lesion. Z Orthop Ihre Grenzgeb.

[7] Rtaimate M, Henry E, Lariviere J, Farez E, Laffargue P (2002). Shoulder fusion for sequelae secondary to brachial plexus palsy. Rev Chir Orthop Reparatrice Appar Mot.

[8] Sousa R, Pereira A, Massada M, Trigueiros M, Lemos R, Silva C (2011). Shoulder arthrodesis in adult brachial plexus injury: what is the optimal position?. J Hand Surg Eur.

[9] Wong EL, Kwan MK, Loh WY, Ahmad TS (2005). Shoulder arthrodesis in brachial plexus injuries-a review of six cases. Med J Malays.

[10] Chammas M, Goubier JN, Coulet B, Reckendorf GM, Picot MC, Allieu Y (2004). Glenohumeral arthrodesis in upper and total brachial plexus palsy. A comparison of functional results. J Bone Joint Surg Br.

[11] Clare DJ, Wirth MA, Groh GI, Rockwood CA (2001). Shoulder arthrodesis. J Bone Joint Surg Am.

[12] Chammas M, Meyer zu Reckendorf G, Allieu Y (1996). Arthrodesis of the shoulder for post-traumatic palsy of the brachial plexus. Analysis of a series of 18 cases. Rev Chir Orthop Reparatrice Appar Mot.

[13] Ducloyer P, Nizard R, Sedel L, Witvoet J (1991). Arthrodesis of the shoulder in paralysis of the brachial plexus. Apropos of 2 cases. Rev Chir Orthop Reparatrice Appar Mot.

[14] Rowe CR (1974). Re-evaluation of the position of the arm in arthrodesis of the shoulder in the adult. J Bone Joint Surg Br.

[15] Hudak PL, Amadio PC, Bombardier C (1996). Development of an upper extremity outcome measure: the DASH (disabilities of the arm, shoulder and hand) [corrected]. The Upper Extremity Collaborative Group (UECG). Am J Ind Med.

[16] Ruhmann O, Schmolke S, Bohnsack M, Kirsch L, Wirth CJ (2004). Shoulder arthrodesis. Indications, techniques, results, complications. Orthopade.

[17] Safran O, Iannotti JP (2006). Arthrodesis of the shoulder. J Am Acad Orthop Surg.

[18] Jonsson E, Lidgren L, Rydholm U (1989). Position of shoulder arthrodesis measured with Moire photography. Clin Orthop Relat Res.

